# Epitranscriptomic Stability—Variable Extents of N1-Methyladenosine to N6-Methyladenosine Conversion Under Different Experimental Conditions

**DOI:** 10.3390/biom16050712

**Published:** 2026-05-12

**Authors:** Frank Morales Shnaider, Hasna Kanan, Shrikant Patel, Norman H. L. Chiu

**Affiliations:** 1 Department of Chemistry and Biochemistry, University of North Carolina Greensboro, 301 McIver St., Greensboro, NC 27412, USA; fams_14@yahoo.com (F.M.S.); h_kanan@uncg.edu (H.K.); sjpatel@uncg.edu (S.P.); 2 Joint School of Nanoscience and Nanoengineering, University of North Carolina Greensboro, 2907 E Gate City Blvd., Greensboro, NC 27401, USA

**Keywords:** epitranscriptome, RNA modifications, stability, quantification

## Abstract

The growing interest in epitranscriptomes has emphasized the need for accurate quantification of RNA modifications. However, the stability of RNA modifications under different experimental conditions remains poorly characterized. In this study, we use N1-methyladenosine (m^1^A) as a model for assessing stability. After exposing m^1^A ribonucleoside to various conditions that are commonly used in RNA protocols, the sample was analyzed using untargeted high-resolution mass spectrometry. Under alkaline or neutral pH, different extents of m^1^A were converted to N6-methyladenosine (m^6^A). Complete m^1^A-to-m^6^A conversion occurred at elevated temperatures. The m^1^A-to-m^6^A conversion was also dependent on the initial m^1^A concentration; the higher the concentration, the higher the rate of conversion. This poses a challenge to comparative studies if the initial amount of m^1^A in the control and sample of interest are not equal. No demethylation or depurination was detected. However, trace amount of N1-methylinosine was detected as a result of non-enzymatic deamination of m^1^A. Furthermore, the m^1^A-to-m^6^A conversion was consistently observed in a biological sample. To eliminate the bias that resulted from m^1^A-to-m^6^A conversion, the standard addition method was adopted. This report highlighted the challenges of having different extents of m^1^A-to-m^6^A conversion under specific experimental conditions and demonstrated a viable solution for resolving the issue.

## 1. Introduction

Following prokaryotic or eukaryotic transcription, RNA molecules may undergo post-transcriptional modifications. The resulting RNA modifications can, in fact, define the identity of a transcript, for instance, the 5′ cap in messenger RNA (mRNA). From the structural point of view, RNA modification(s) can potentially induce or alter RNA folding [1,2]. In the case of transfer RNA (tRNA), the co-existence of multiple RNA modifications on the same RNA molecule is needed to stabilize its cloverleaf structure. As many as 170 RNA modifications have been reported in the literature [3]. Among the known RNA modifications, only a few modifications are well characterized, which include pseudouridine, inosine, and N6-methyladenosine (m^6^A), while the functions of many other RNA modifications remain largely unknown [4]. With the discovery of specific enzymes for adding or removing RNA modifications, and specific proteins that recognize RNA modifications, the study of RNA modifications and their associated molecular networks has led to the emergence of another omics field, called epitranscriptomes [5,6]. Recently, the variations in m^6^A level have been found to associate with different types of cancer [7,8]. Hence, one of the current efforts in studying epitranscriptomes is how specific RNA modifications regulate cellular activities. To achieve that, high accuracy in the identification and quantification of targeted RNA modifications is crucial.

**Figure 1:**
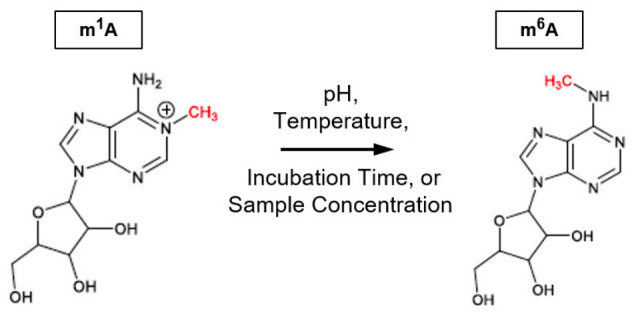
Schematic diagram showing the m^1^A to m^6^A conversion under various experimental conditions.

Traditionally, the majority of RNA modification analyses have been conducted after RNA was extracted from cellular materials, rather than within the cells. During the process of RNA extraction, RNA is exposed to conditions at different pHs, disregarding which protocol was chosen [9,10]. Despite the extra time and labor required for RNA extraction, the cell-free approach is preferred because higher detectability of RNA modifications can be achieved, which is essential for studying low abundance RNA modifications. For the detection of specific RNA modifications, according to a recent report released by the National Academy of Sciences of the United States of America [6,9], the current technologies can be divided into three categories, namely next generation sequencing, nanopore sequencing, and mass spectrometry. Prior to any sequencing methods, RNA folding and intermolecular RNA-RNA duplexes must be denatured, which requires exposing RNA samples to an elevated temperature (≥65 °C). To facilitate the detection of RNA modifications, other necessary steps in the protocols may include reverse transcription or RNA digestion. As a result, the RNA samples are further exposed to elevated temperatures and different pHs. Although there were reports on the effects of elevated temperatures or pHs on RNA samples, the impact of those experimental parameters on RNA modifications remains largely unknown [11]. This is because many ribonucleoside standards are either not commercially available or priced beyond the budgetary constraints of many research labs. If specific RNA modifications are degraded during the steps of sample preparation, the results from determining the amounts of RNA modifications would become inaccurate. To demonstrate the chemical stability of specific RNA modifications is crucial to epitranscriptomic studies especially when quantitative data are involved, we have selected N1-methyladenosine (m^1^A) as our model of RNA modification. The m^1^A modification has been detected in a variety of RNA molecules, including mRNA, tRNA, and ribosomal RNA [11,12,13,14]. According to earlier reports [15,16], under alkaline pH conditions, the positively charged m^1^A molecule reacts readily with the hydroxyl ion, which results in the relocation of the methyl group at the N^1^ position to the N^6^ position of adenosine (Figure 1). In other words, m^1^A can potentially be converted into N6-methyladenosine (m^6^A), which is often recognized to be the most frequent RNA modification and used extensively as an epitranscriptomic biomarker [4,7,8]. Despite the fact that the m^1^A-to-m^6^A conversion is known, the extent of conversion under specific experimental conditions remains largely unknown. Therefore, if protocols with different experimental conditions are adopted to analyze a specific epitranscriptome, it will be more challenging to correlate the results and/or associate the variations in m^1^A or m^6^A level to specific diseases [17,18,19,20,21,22,23]. To address these challenges, this report focuses on determining the extent of m^1^A to m^6^A conversion under various experimental conditions that are commonly used in RNA or epitranscriptomic studies; and explores a simple yet practical method for determining the original m^1^A concentration in samples before its conversion to m^6^A.

## 2. Materials and Methods

### 2.1. Effects of Neutral pH

m^1^A ribonucleoside standard (0.11 µM) from Biosynth (Compton Berkshire, UK) was freshly prepared in Optimal grade HPLC water (ThermoFisher, Waltham, MA, USA), which was measured at pH 7. For the control, the reported alkaline condition for m^1^A to m^6^A conversion was used, in which the m^1^A standard was prepared in 50 mM Tris-HCl buffer, pH 8. All incubation was carried out in a thermocycler. An aliquot was removed from each m^1^A solution after 1, 3, 6, and 12 h of incubation, and the analysis of both m^1^A and m^6^A was performed as described in Reference [24]. A 10 µL sample was injected onto a an Acquity ultra-performance liquid chromatography (UPLC) system (Waters Corporation, Milford, MA, USA) with a binary pump and an autosampler maintained at 4 °C. A Water’s Acquity UPLC HSS T3 column (2.1 × 50 mm, 1.8 µm particle size) and a HSS T3 VanGuard pre-column (2.1 mm × 5 mm, 1.8 µm particle size) with an Acquity inline filter kit held at 30 °C were used. The elution was carried out with a two-solvent system prepared with Optima grade solvents. Mobile Phase A consisted of H_2_O with 0.01% formic acid and Mobile Phase B consisted of 50% CH_3_CN with 0.01% formic acid held at a flow rate of 0.4 mL/min. The binary elution gradient was carried out as follows: an initial isocratic composition of 100:0 (A:B) from 0.0 to 0.5 min., increasing to 70:30 from 0.5 to 9.0 min. at a curve of 7, linearly changing to 50:50 from 9.0 to 10.0 min., followed by another linear increase to 0:100 from 10.0 to 15.0 min., a final isocratic hold at 0:100 from 15.0 to 16.0 min. marks the transition to column wash and re-equilibration at the starting conditions for 8 min. High resolution tandem mass spectrometry (MS/MS) was performed on a Q Exactive Plus mass spectrometer (ThermoFisher Scientific, Waltham, MA, USA) in the positive mode with a heated electrospray ionization source (HESI) with heater temperature at 425 °C, capillary temperature 275 °C, and spray voltage at 3.5 kV. Sheath and auxiliary gas flow were set at the arbitrary values of 50 and 13, respectively. Mass spectra were collected using two scan events. The first scan was a full scan between 100 and 700 m/z at a resolution of 70,000. The second scan employed an inclusion list of the calculated m/z of all known RNA modifications as reported in the Modomics database with a 1.0 m/z isolation window. A two-step higher-energy collisional dissociation (HCD) fragmentation was employed at normalized collision energies (NCE) of 20 and 110 at 17,500 resolution. The goal was to achieve the detection of at least two corresponding fragment ions. Prior to each experiment, mass calibration was performed using a canonical ribonucleoside standard mixture (3.25 µM) with an error of <1 ppm and retention time accuracy ± 0.1 min.

Each RNA sample was analyzed in triplicate, and blanks were used between repeated measurements. If multiple RNA samples were analyzed in the same experiment, the order of the repeated measurements was randomized. For the purpose of detecting any unexpected m^1^A degradation, a data-independent approach was used to acquire the MS signals. At the end of each analysis, the UPLC system was rinsed. Each MS signal was normalized to the sum of signals, and the average signal was used to calculate the relative amount of m^1^A.

For quantitative analysis of UPLC–MS data, the raw data were processed using Xcalibur (Version 3, ThermoFisher, Waltham, MA, USA). Specifically, extracted ion chromatograms were generated based on the protonated molecular ions of m^1^A and m^6^A. The corresponding peak areas were obtained from each replicate analysis. Peak assignment was accepted only when both the retention time and accurate mass matched the expected values within predefined tolerances of ±0.2 min and ±5 ppm, respectively.

For each condition, analyses were performed in triplicate, and the average peak area was calculated. The relative abundance of m^1^A or m^6^A was then determined by normalizing the average peak area of each analyte to the sum of the average peak areas of m^1^A and m^6^A, thereby accounting for variations in signal intensity between runs.

### 2.2. Effects of Incubation Temperature

For each selected temperature, this was conducted with a set of 7 identical aliquots, one of the aliquots was kept frozen at −20 °C, which served as a control with no exposure to any elevated temperature. The sample was freshly prepared with m^1^A standard at 0.20 µM in 50 mM Tris-HCl buffer, pH 8. All incubation was carried out simultaneously in a thermocycler. The longest duration for incubating the sample was lowered from 48 h to 24 h when the incubation temperature was increased to 57 °C. At each time point, an aliquot was removed and analyzed as described above.

### 2.3. Effects of m^1^A Concentration

m^1^A samples at different concentrations were freshly prepared in 50 mM Tris-HCl, pH 8. The samples were incubated at 37 °C for 12 h. After the incubation, the samples were analyzed as described above.

### 2.4. Effects of Sample Complexity

An epitranscriptomic sample originated from a well-characterized glioblastoma (GBM) cell line (LNZ-308) was prepared as reported in our earlier studies [24]. Briefly, the GBM sample contained all the unmodified and modified ribonucleosides. In the latter case, it included 31 different types of RNA modification, which included both m^1^A and m^6^A. The level of m^1^A in the GBM sample was determined using a standard curve. Three aliquots of the GBM sample (500 ng, pH 8) with equal volume were used. To the first aliquot, no m^1^A standard was added. To the second aliquot, a known amount of m^1^A standard was added, which was equal to the same amount of m^1^A as determined in the GBM sample. To the third aliquot, four times the amount of m^1^A standard was added. After that, deionized water was used to make up the total volume in each aliquot to be identical in all three aliquots. A control with only m^1^A standard at the same level as in the GBM sample was included. All four samples were incubated at 37 °C for 12 h. At the end of the incubation, each sample was analyzed as described above.

### 2.5. Determination of Original m^1^A Concentration Using the Standard Addition Method

To determine the original m^1^A concentration in a biological sample, the standard addition method was adopted. Specifically, the sample of interest was first divided into at least three equal aliquots. No m^1^A standard was added to the first aliquot. To the remaining aliquots, known amounts of m^1^A standard were added in increasing quantities while ensuring that the total amount of m^1^A remained within the established linear dynamic range of the m^1^A measurements (10–15,000 pg). Based on our earlier study, the endogenous level of m^1^A in the selected biological sample was estimated to be approximately 500 pg. Therefore, 1000 pg and 1500 pg of m^1^A standard were added to the second and third aliquots, respectively. The total volume of each aliquot was adjusted to be identical by adding an appropriate amount of RNase-free deionized water. After that, all the mixtures were treated equally and incubated at 37 °C for 12 h. The signal of m^1^A acquired from each mixture was then plotted against the amount of m^1^A standard added. After performing the linear regression analysis, the best fit line was extrapolated. The original m^1^A concentration in the sample of interest is equal to the crossing on the *x*-axis (Figure 5b).

## 3. Results

### 3.1. Conversion of m^1^A → m^6^A at Neutral pH

Since the m^1^A-to-m^6^A conversion under alkaline conditions and the depurination of nucleotides under acidic conditions have been reported [18], our initial efforts focused on determining whether the extent of m^1^A-to-m^6^A conversion under neutral pH would be significant, i.e., detectable. If so, an in-house mass spectrometric method recently developed in our group would be used to accurately measure the extent of m^1^A-to-m^6^A conversion over a period of incubation time [24]. It is important to point out that RNase-free water at neutral pH is commonly used to store RNA samples. To perform this study, pure m^1^A ribonucleoside standard was used as the starting material, which was diluted in deionized water tested to be at pH 7. Prior to studying the conversion, the m^1^A standard was tested to be free of contaminants, thus allowing us to detect any unexpected degradation of m^1^A. Another sample with the same amount of m^1^A standard but diluted in 50 mM of Tris at pH 8 was used as a positive control for the m^1^A-to-m^6^A conversion. For the identification of m^1^A or m^6^A, reversed-phase liquid chromatography coupling to high resolution mass spectrometry was used and baseline chromatographic resolution was achieved. To facilitate the detection of any m^1^A degradation, an untargeted data-independent method was set up to scan a mass window of 100 to 1000 m/z during the MS measurements [25]. To achieve accurate quantitative measurements, ribonucleoside standards were used to establish the limit of detection with a signal-to-noise >2 and a linear dynamic range of >3 orders of magnitude as well as an average coefficient of variation of ~10% as reported in our earlier study [24]. It is important to note that the MS signals of m^1^A and m^6^A should not be used directly to calculate the extent of m^1^A-to-m^6^A conversion. This is because the efficiency of generating the molecular ions of m^1^A and m^6^A in the first step of the MS measurement, namely electrospray ionization, is not equal to each other. Therefore, after any m^1^A-to-m^6^A conversion has taken place, the decrease in the m^1^A signal does not match the increase in the m^6^A signal. Consequently, the extent of conversion should not be determined simply by comparing the relative peak intensities of m^1^A and m^6^A in the mass spectra. To overcome this issue, instead of calibrating the MS signals with a series of standard dilutions, our in-house MS method was designed to correct the difference in MS ionization efficiency [24]. An alternative approach for quantifying the amount of m1A and m6A is to perform MS calibration using a series of appropriate standard dilutions.

**Figure 2:**
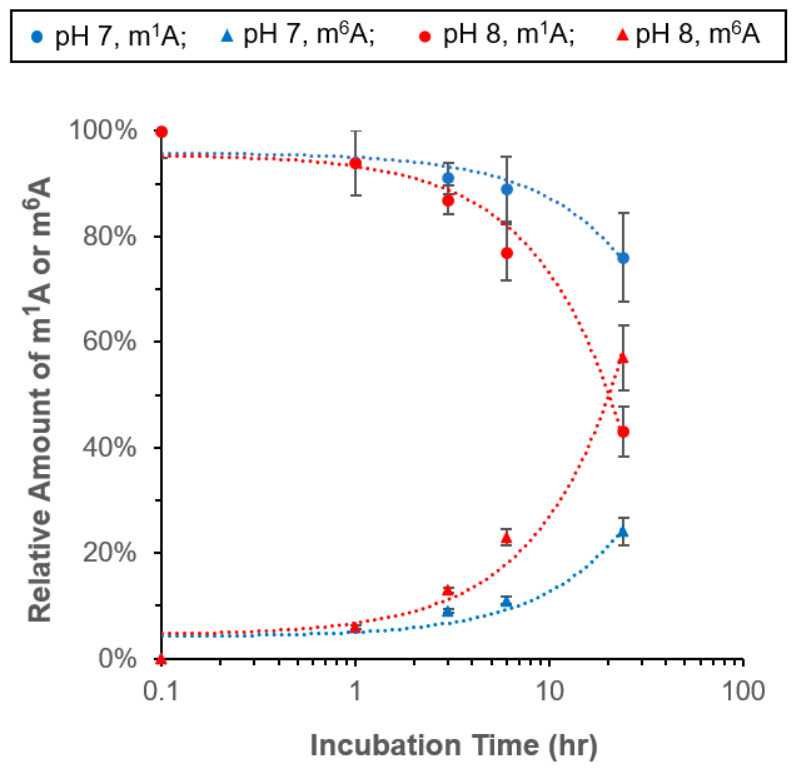
Effects of neutral pH on the chemical stability of m^1^A. An amount of 0.11 µM of m^1^A ribonucleoside standard was incubated in water (pH 7) at 37 °C for an extended period of time. The relative amount of m^1^A is referenced to the initial amount of m^1^A. Error bars represent the coefficient variation in repeated measurements (n = 3).

The results from exposing m^1^A to neutral pH are shown in Figure 2. At 37 °C incubation, the extent of m^1^A → m^6^A conversion at pH 7 increases exponentially with increasing incubation time. It follows the same trend as the positive control at pH 8. At pH 7, after the m^1^A sample was incubated for 1 h, 6% of the original amount of m^1^A was converted to m^6^A. The percent of conversion (or relative amount) was calculated from the average MS signals of m^1^A before and after the incubation. The answer was then cross checked with the result obtained from measuring m^6^A. Furthermore, the MS signal of m^1^A or m^6^A was identified by matching its retention time, accurate molecular mass, and the presence of the corresponding fragment ions in the MS/MS measurements. The percent of conversion was increased to 9% after 3 h of incubation at pH 7. When the incubation was extended to 12 h, as much as 24% of m^1^A was converted while no depurination was detected. These results demonstrate that the m^1^A → m^6^A rearrangement can occur even under conditions that are commonly considered chemically mild for RNA handling.

In comparison to the results obtained from incubating the m^1^A standard at pH 8, the overall rate of converting m^1^A to m^6^A at pH 7 is relatively low unless the incubation time is longer than ~3 h (blue vs. red dotted line in Figure 2). This is because the concentration of hydroxyl ions at pH 7 is one order of magnitude lower than that at pH 8. This finding is consistent with the base-catalyzed nature of the Dimroth rearrangement, in which a higher hydroxyl ion concentration promotes faster conversion of m^1^A to m^6^A.

### 3.2. Temperature-Dependent Conversion of m^1^A → m^6^A

**Figure 3:**
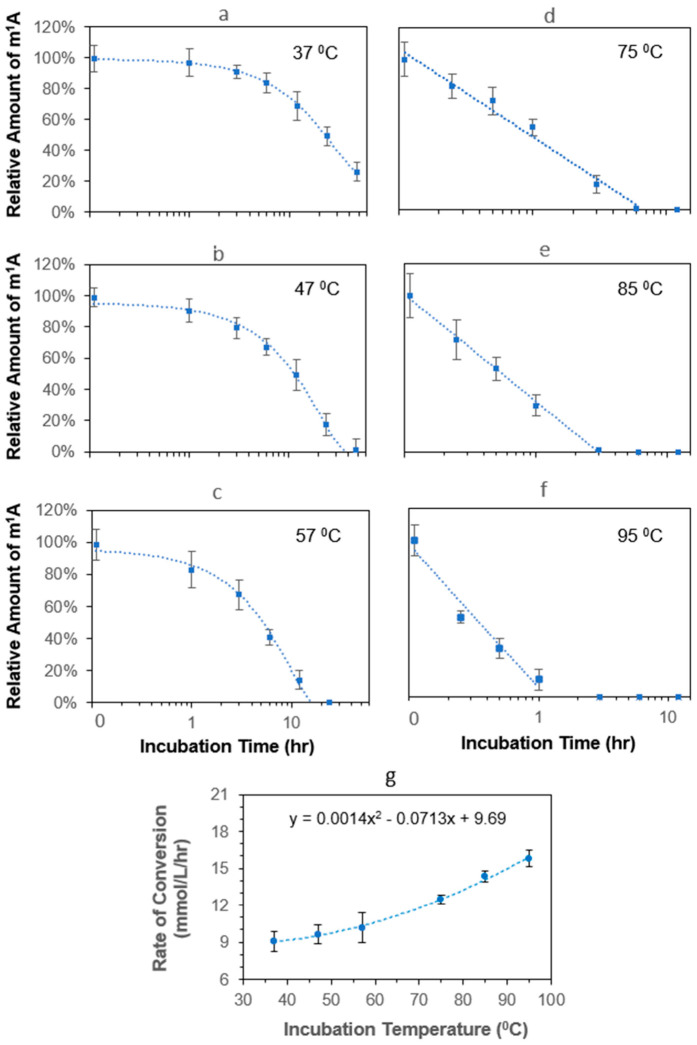
Effects of temperature on the chemical stability of m^1^A. (**a**–**f**) An amount of 0.20 µM of m^1^A ribonucleoside standard, which was diluted in 50 mM of Tris (pH 8), was incubated at selected temperatures over a time course of 24 or 48 h. The relative amount of m^1^A is referenced to the initial amount of m^1^A. Data points at zero percent represent that no m^1^A was detected. The trendline (excluding the zero data points) is inserted. Each error bar represents one standard deviation of repeated measurements (n = 3). (**g**) Scatter plot showing the average rate of converting m^1^A to m^6^A increases exponentially with incubation temperature. The rates of conversion are based on the data shown in (**a**–**f**).

Owing to the inter/intramolecular interactions of RNA, the use of an elevated temperature to denature RNA in a protocol for RNA analysis is a very common practice, plus no data on the m^1^A conversion at temperatures above 37 °C is currently available [10], these prompted us to carry out a comprehensive study that aimed at covering the entire range of possible temperatures (37 to 95 °C) used in different protocols [26,27,28]. To simulate the conditions in those RNA protocols, neutral pH was not chosen. Instead, the m^1^A standard was prepared in Tris buffer at pH 8. The selected range of temperatures is divided into two groups as shown in Figure 3a–c and Figure 3d–f. In each group, the temperature is increased by an increment of 10 °C. To ensure this study would cover the conditions used in various protocols, the incubation time at each selected temperature was extended to the point when the majority of m^1^A was converted to m^6^A. To reduce the effects of concentrating the sample caused by evaporation during the retrieval of sample at each time point shown in Figure 3a–f, the original m^1^A standard solution was divided into separate microcentrifuge tubes before starting the incubation. The results from incubating m^1^A at 37 °C indicate the percent of m^1^A conversion increases exponentially over 48 h (Figure 3a). After 48 h of incubation, only ~20% of m^1^A remained or ~80% of m^1^A was converted to m^6^A. The same percentage of m^1^A conversion was achieved in about half the incubation time (24 h) when the temperature went up to 47 °C (Figure 3b). A similar outcome was obtained from the incubation at 57 °C (Figure 3c). In Figure 3, zero percent of m^1^A represents no m^1^A was detected. When the incubation temperature was increased from 57 °C to 75 °C, the same level of m^1^A conversion (80%) was accomplished in about 3 h (Figure 3d). The time required to reach the same level of m^1^A conversion lowered to less than 1 h when the incubation temperature maxed out at 95 °C (Figure 3f). Taken together, these data show that the conversion of m^1^A to m^6^A becomes markedly accelerated as the incubation temperature increases, especially above 57 °C. The sharp reduction in the time required to achieve extensive conversion at 75–95 °C demonstrates that high-temperature incubation steps commonly used in RNA protocols can substantially alter the measured level of m^1^A within a relatively short time. This implies even 5–10 min sample incubation at 95 °C, for example the step in which total RNA is denatured prior to the annealing of an oligo primer in the process of reverse transcription, would convert a significant amount of m^1^A to m^6^A. If the m^1^A modification happens to be located within the position where the oligo primer is expected to anneal, the conversion of m^1^A to m^6^A would theoretically increase the annealing temperature. This is because the methyl group at the N1 position would disrupt the hydrogen bonding to uracil. Whereas, the methyl group at the N6 position would have the opposite effect.

Based on the data in Figure 3a–f with 1 h of incubation, we calculated the rate of m^1^A conversion (Figure 3g). At 37 °C, the rate of m^1^A conversion started from 9.1 mmol/L/h and increased slowly to 10.2 mmol/L/h after the incubation temperature was increased to 57 °C. However, if the standard deviations, i.e., error bars in Figure 3g, are taken into consideration, there is no significant change in the rate of m^1^A conversion within the first half of the curve in Figure 3g (37 °C → 57 °C). In the second half of the curve in Figure 3g (75 °C → 95 °C), the rate of m^1^A conversion increases more sharply with increasing temperature, reaching 15.8 mmol/L/h at 95 °C. These results show the conversion of m^1^A to m^6^A is temperature dependent. If RNA samples are exposed to elevated temperatures, the incubation time required to convert a fixed amount of m^1^A is decreased exponentially. The data in Figure 3g further indicate that the temperature effect is not linear across the full range studied. Rather, the influence of temperature is modest between 37 and 57 °C but becomes much more pronounced at 75 °C and above, indicating that the chemical stability of m^1^A deteriorates rapidly under conditions with higher temperature. By using the quadratic equation inserted in Figure 3g, the extent of m^1^A conversion results from exposing an RNA sample to a specific temperature can be estimated, providing the incubation time and pH value are equal to one hour and pH 8, respectively. To the best of our knowledge, this is the first time such information is reported.

When monitoring the conversion of m^1^A to m^6^A by using an untargeted MS method, it renders us an opportunity to determine whether additional conversion of m^1^A or the degradation of m^1^A occur concurrently during the prolonged incubation of m^1^A at elevated temperatures. In this regard, the demethylation of m^1^A to adenosine was never detected. On the other hand, a trace amount of m^1^I was detected whose identity was confirmed by accurate mass measurement of protonated m^1^I ion, and its matching fragment ions (Supplemental Figure S1a,b). To the best of our knowledge, this is also the first report on detecting a non-enzymatic conversion of m^1^A to m^1^I.

### 3.3. Concentration-Dependent Conversion of m^1^A → m^6^A

The last but the most important experimental condition being investigated in this report is the sample concentration. Specifically, the sample concentration is defined here as the amount of m^1^A in the original sample before any of them is chemically converted to m^6^A. Depending on the dynamic activities of the corresponding writers and erasers for m^1^A modification, some of the adenosine in specific transcripts may be modified while the other adenosine remains unmodified. In other words, different biological samples would have different amounts of m^1^A. For instance, untreated cellular samples (or controls) in a biomedical study may have different amounts of m^1^A in comparison to the treated samples. If so, the approach of normalizing the average signals of m^1^A acquired from the treated samples to the average signals obtained from the untreated samples is only correct if the conversion of m^1^A to m^6^A is NOT concentration dependent.

**Figure 4:**
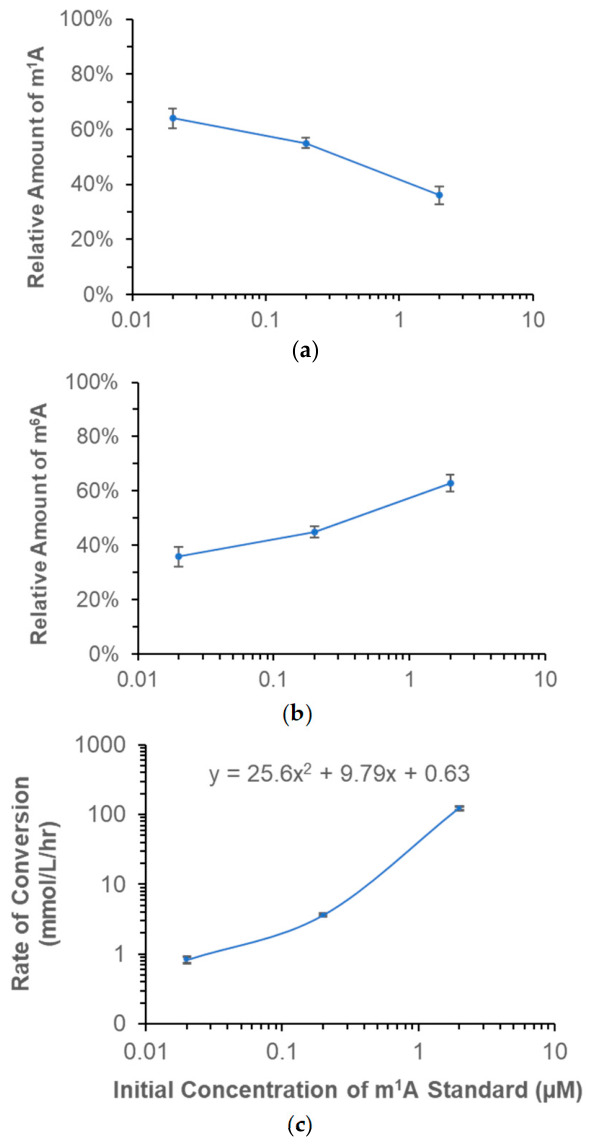
(**a**,**b**) Effects of m^1^A concentration on its chemical stability. Various concentrations of m^1^A ribonucleoside standard were incubated at 37 °C for 12 h. Each standard solution was diluted in 50 mM of Tris at pH 8. The relative amount of m^1^A (or m^6^A) is referenced to the initial amount of m^1^A. (**c**) Scatter plot showing the rate of converting m^1^A to m^6^A increases with the initial m^1^A concentration. Error bars represent the coefficient variation in repeated measurements (n = 3).

To determine the effects of m^1^A concentration on the conversion of m^1^A to m^6^A, we first determined the average level of m^1^A in different human cell lines that were available in our lab. Then, with the knowledge of the linear dynamic range in the calibration curve established using m^1^A standard dilutions, we extended the range of m^1^A concentration accordingly from the average value of m^1^A concentration detected in the biological samples. This resulted in a range of m^1^A concentration, spanning from 0.02 to 2 µM, for our study on the concentration effects. In Figure 4a, it clearly shows the conversion of m^1^A to m^6^A is concentration dependent. The higher the m^1^A concentration, the higher the percent of conversion. Specifically, when the m^1^A concentration was at 2 µM, only 36% of m^1^A would remain unchanged after an overnight incubation at 37 °C and pH 8. The results of m^1^A conversion are confirmed by analyzing and plotting the results obtained from measuring m^6^A in Figure 4b, which turns out to be a mirror image of Figure 4a. Although these results were obtained from an overnight incubation, the condition is comparable to some of the enzymatic reactions used in the current protocols for RNA analysis [29,30,31].

Based on the data in Figure 4a, the rate of m^1^A conversion was calculated and shown in Figure 4c. At the lowest m^1^A concentration of 0.02 µM, the conversion rate was only 0.8 mmol/L/h. When m^1^A concentration was increased 10 times to 0.2 µM, the conversion rate went up to 3.6 mmol/L/h, which represents a more than 4-fold increase in the conversion rate. Furthermore, when m^1^A concentration was increased from 0.2 to 2 µM, the conversion rate reached 123 mmol/L/h, which represents a 34-fold increase. In comparison to the conversion rates at different temperatures (Figure 3g), the conversion rates associated with increasing m^1^A concentration are significantly higher. In other words, between the effects of temperature and concentration, the conversion of m^1^A to m^6^A is mainly driven by the m^1^A concentration. To the best of our knowledge, this finding has not been reported before in the literature.

### 3.4. Conversion of m^1^A → m^6^A in Biological Sample

**Figure 5:**
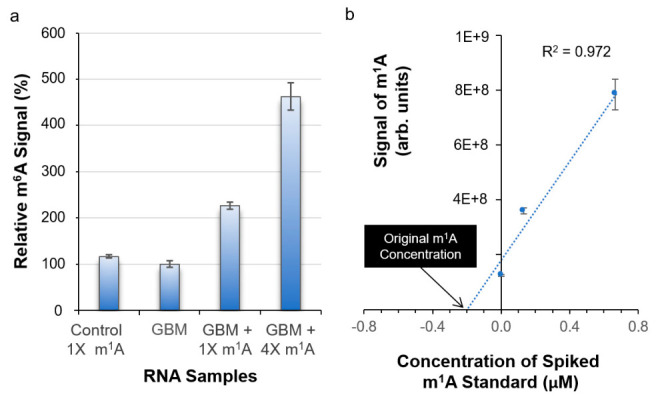
(**a**) Bar chart showing the results from spiking a glioblastoma (GBM) digested RNA sample with known amounts of m^1^A ribonucleoside standard. 1X is equivalent to 0.17 μmol. (**b**) Scatter plot showing how the original m^1^A concentration can be experimentally determined by adopting the standard addition method. Error bars represent the standard deviation of repeated measurements (n = 3).

In our ongoing related epitranscriptomic studies, one of the diseases we have focused on is glioblastoma (GBM). Using the mass spectrometric method to profile GBM epitranscriptomes, the identity and quantity of each detectable RNA modification were determined. In total, 31 different RNA modifications were detected, which included both m^1^A and m^6^A [24]. To assess whether our findings on the conversion of m^1^A standard to m^6^A are applicable to biological samples, we spiked known amounts of m^1^A standard into a GBM sample containing a pre-determined m^1^A level. When an equal amount of m^1^A standard (1X) was added, effectively doubling the total amount of m^1^A in the mixture, the resulting m^6^A signal after an incubation at 37 °C for 3 h was approximately doubled (Figure 5a). This was repeated by increasing the amount of m^1^A spiking to 4X, and the m^6^A signal acquired from the mixture was found to be increased proportionally (Figure 5a). Therefore, despite the presence of many other modified and unmodified ribonucleosides in the GBM sample, the conversion of m^1^A to m^6^A proceeds in a consistent manner.

### 3.5. Determining Original m^1^A Concentration

**Figure 6:**
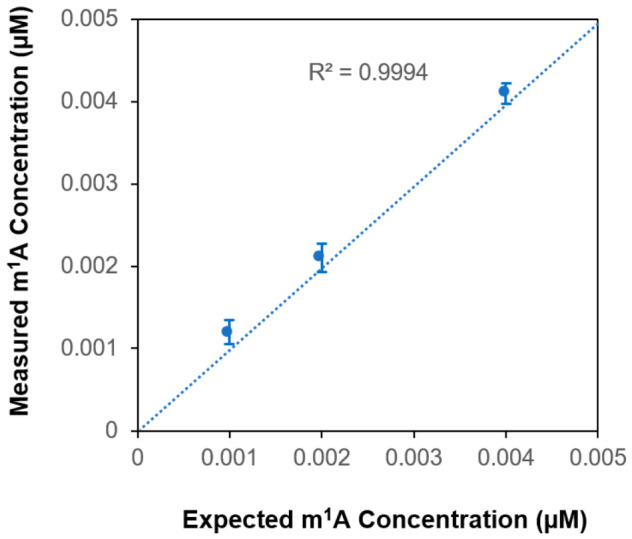
The measured m^1^A concentrations which are determined using the standard addition method are plotted against the expected m^1^A concentration. Error bars represent the standard deviation of repeated measurements (n = 3). R^2^ = (correlation coefficient)^2^.

When determining an unknown concentration of a selected analyte in a sample with a complex matrix, the other sample components may interfere with the analyte measurement and cause the signal to go up or down. As a result, it prohibits the use of a standard curve to determine the unknown concentration, unless the standard dilution could be carried out with a diluent containing the same complex matrix as in the sample of interest. An effective way to overcome this problem is by using the standard addition method [32]. By considering the result from converting m^1^A to m^6^A under a specific experimental condition as an equivalence to suppressing the m^1^A signal, we applied the standard addition method to determine the original m^1^A concentration in the GBM sample, i.e., before any m^1^A is converted to m^6^A. Specifically, the GBM sample was divided into three aliquots, one without any spiking of m^1^A standard while the other two aliquots were spiked with increasing and known amount of m^1^A standard. After processing the three mixtures as described in our established LC-MS protocol, the m^1^A signal was acquired from each mixture and plotted against the amount of spiked m^1^A standard (Figure 5b). By extrapolating the data points in Figure 5b, the line crossing the *x*-axis is equal to the original m^1^A concentration in the GBM sample, which was determined to be 0.2 µM. To validate the use of the standard addition method to determine the original m^1^A concentration, we carried out a correlation study. By considering three different m^1^A standard dilutions as unknown samples, we applied the standard addition method to determine the m^1^A concentration. The measured m^1^A concentration was plotted against the expected m^1^A concentration (Figure 6), and the two sets of m^1^A concentrations correlate well with each other with a correlation coefficient of 0.9994, thus showing that the standard addition method is a viable approach to determine the original m^1^A concentration in a sample of our interest. For samples with limited RNA materials, the use of a more sensitive endpoint detection method, such as triple-quadrupole LC–MS operated in multiple-reaction monitoring mode, may facilitate standard addition by allowing the sample to be diluted and divided into the required aliquots while maintaining detectability.

If the research interest of a future study focuses on the quantity of m^6^A instead of m^1^A, the original or correct m^6^A concentration without any biases that result from m^1^A → m^6^A conversion can be calculated using the results acquired from the standard addition method. Specifically, by assuming the conversion of m^1^A to m^6^A occurs exclusively in a 1:1 ratio, and after determining the original m^1^A concentration as described above, the difference between the original m^1^A quantity and the decreased level of m^1^A would be equal to how much of the m^6^A quantity is increased.

## 4. Conclusions

When using m^1^A as a quantitative biomarker, the non-enzymatic conversion of m^1^A to m^6^A under various experimental conditions represents a challenge to epitranscriptomic or related studies. Although the m^1^A conversion is unavoidable when handling the RNA samples in an aqueous environment, maintaining the pH at 7 would minimize the conversion. No depurination and demethylation of m^1^A were detected even with extended incubation at elevated temperatures, but a trace amount of m^1^I was identified as a product of non-enzymatic deamination of m^1^A. For the first time, the standard addition method was applied as a solution for determining the correct m^1^A concentration in a sample of interest. This approach can potentially be extended to remove the biases in measuring the quantity of m^6^A.

## Data Availability

The datasets analyzed during the current study are available from the corresponding author upon reasonable request.

## Supplementary Materials

The following supporting information can be downloaded at: https://www.mdpi.com/article/10.3390/biom16050712/s1, Figure S1. (a) Extracted ion chromatogram of the positively charged 1-methylinosine (m^1^I) with 1.06 ppm mass accuracy, which was acquired from analyzing the degradation of m^1^I ribonucleoside standard after 24 h of heat treatment at 95 °C. (b) MS/MS spectrum of the precursor m^1^I ion.
