# Supplementary material for: Epitranscriptomic Stability—Variable Extents of N1-Methyladenosine to N6-Methyladenosine Conversion Under Different Experimental Conditions
Source: Biomolecules. 2026 May 12;16(5):712. doi: 10.3390/biom16050712 (PMC13204903; doi:10.3390/biom16050712)
Supplement: Supplementary file 1 [file biomolecules-16-00712-s001.zip › biomolecules-4155690-supplementary.pptx]

## Slide 1
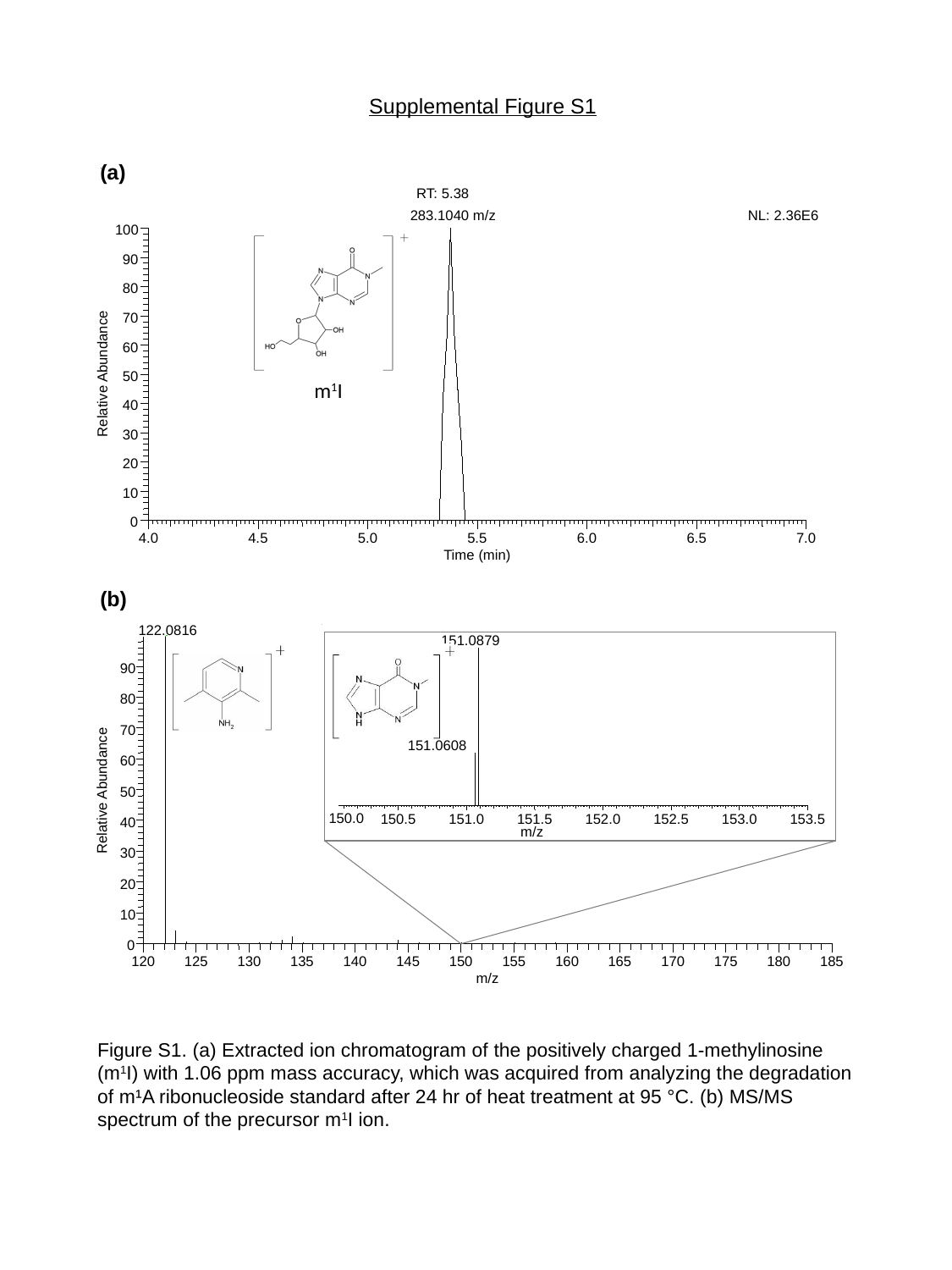

Supplemental Figure S1
(a)
4.0
4.5
5.0
5.5
6.0
6.5
7.0
Time (min)
RT: 5.38
283.1040 m/z
NL: 2.36E6
100
90
80
70
60
Relative Abundance
50
40
30
20
10
0
m1I
(b)
122.0816
90
80
70
60
Relative Abundance
50
40
30
20
10
0
120
125
130
135
140
145
150
155
160
165
170
175
180
185
m/z
150.5
151.0
151.5
152.0
152.5
153.0
153.5
m/z
151.0879
150.0
151.0608
Figure S1. (a) Extracted ion chromatogram of the positively charged 1-methylinosine (m1I) with 1.06 ppm mass accuracy, which was acquired from analyzing the degradation of m¹A ribonucleoside standard after 24 hr of heat treatment at 95 °C. (b) MS/MS spectrum of the precursor m1I ion.
